# In Vitro Purging of Acute Lymphoblastic Leukemia (B-ALL) Cells with the Use of PTL, DMAPT, or PU-H71

**DOI:** 10.3390/ijms252111707

**Published:** 2024-10-31

**Authors:** Ana Elenka Ortiz-Reyes, Sergio García-Sánchez, Montserrat Serrano, Juan Carlos Núñez-Enriquez, José Antonio Alvarado-Moreno, Juan José Montesinos, Guadalupe Fajardo-Orduña, Monica L. Guzman, Miguel Angel Villasis-Keever, Ismael Mancilla-Herrera, Hector Mayani, Antonieta Chavez-Gonzalez

**Affiliations:** 1 Laboratorio de Células Troncales Tumorales, Unidad de Investigación Médica en Enfermedades Oncológicas, CMN Siglo XXI, Instituto Mexicano del Seguro Social, CDMX 06725, Mexico; 2 División de Investigación en Salud, UMAE Hospital de Pediatria, CMN Siglo XXI, IMSS, CDMX 06725, Mexico; 3 Unidad de Investigación Médica en Trombosis Hemostasia y Aterogenesis, Instituto Mexicano del Seguro Social, CDMX 03100, Mexico; 4 Laboratorio de Células Troncales Mesenquimales, Unidad de Investigación Médica en Enfermedades Oncológicas, CMN Siglo XXI, Instituto Mexicano del Seguro Social, CDMX 06725, Mexico; 5 Divivsion of Hematology and Oncology, Department of Medicine, Weill Cornell Medical College, New York, NY 10065, USA; 6 Unidad de Investigación en Análisis y Síntesis de Evidencia, Instituto Mexicano del Seguro Social, CDMX 06725, Mexico; 7 Departamento de Infectología, Instituto Nacional de Perinatología Isidro Espinosa de los Reyes, CDMX 11000, Mexico; 8 Laboratorio de Células Troncales Hematopoyéticas, Unidad de Investigación Médica en Enfermedades Oncológicas, CMN Siglo XXI, Instituto Mexicano del Seguro Social, CDMX 06725, Mexico

**Keywords:** acute lymphoblastic leukemia, leukemic initiating cells, parthenolide, small molecules

## Abstract

Acute lymphoblastic leukemia (ALL) is a hematopoietic disorder that mainly affects the child population, and it is characterized by the presence of lymphoid progenitor or precursor cells with different genetic alterations. The origin of this disease is controversial, since some authors assumed that leukemic transformation occurs in a lymphoid progenitor, and there is also evidence that suggests the existence of leukemic initiating cells (LIC). PTL, DMAPT, and PU-H71 are agents that have been shown to eliminate bulk and stem cells from myeloid leukemias, but this effect has not been analyzed in lymphoblastic leukemias. In this study, we evaluated the effect of these compounds in different populations from pediatric B-ALL. For this, bone marrow samples from pediatric patients without treatment were obtained and cultured in the presence or absence of PTL, DMAPT, and PU-H71. The viability and apoptosis index were analyzed by flow cytometry in different hematopoietic subpopulations. These observations indicate that PTL and DMAPT are able to reduce B-ALL cells with a minimum effect in normal hematopoietic and non-hematopoietic cells. In contrast, PU-H71 was able to reduce the leukemic population and had a minimal effect in normal cells. These results present evidence that PTL and DMAPT are able to abrogate in vitro different populations of B-ALL and could represent a possibility of treatment, as well as prevent disease progression or relapse.

## 1. Introduction

B-cell acute lymphoblastic leukemia (B-ALL) represents a heterogeneous hematological disorder characterized by abnormal proliferation, prolonged survival, and altered differentiation of B-cell precursors or hematopoietic progenitors in the bone marrow, peripheral blood, and extramedullary sites [1]. Some acute lymphoblastic leukemia (ALL) patients show numerical or structural chromosome alterations at diagnosis, and a small fraction of leukemic cells seem to be able to initiate ALL in mice immunodeficient models; this capability has been associated with the presence of leukemia-initiating cells (LIC) that show specific immunophenotypic characteristics [2,3] or particular metabolic activity [4].

Currently, ALL patients are treated with chemotherapeutic agents that include a combination of anthracycline, vincristine, alkylating agent, and corticosteroid that are administrated in remission induction, consolidation, intensification, and maintenance phases where they are able to eliminate the majority of leukemic blasts [5]; however, they fail to eliminate LIC, which appear to be quiescent, have the ability to remodel their niche, are responsible for resistance to treatments, and are responsible for relapse [6]. Thus, the evaluation of novel molecules that could eliminate all leukemic populations is one of the goals in ALL investigation.

Parthenolide is a traditional medicinal plant used for digestive, arthritic, and coagulation affections, and its effect on the hematopoietic system has shown it eradicates total and primitive (stem and progenitor) cells from acute myeloid leukemia (AML) and chronic myeloid leukemia (CML), an effect shared with its analog dimethylamino-parthenolide (DMAPT), which represents a more soluble derivative [7]. Both molecules can inhibit the activity of NFkb transcription factor and induce reactive oxygen species while having no toxic effect in total or stem cell populations from normal hematopoietic samples [8,9,10]. Conversely, PU-H71 is a heat shock protein 90 kDa (Hsp90) inhibitor capable of binding HSP90 chaperones to form a complex with oncogenic molecules that are involved with the malignant phenotype [11]. Moreover, PU-H71 has been shown to reduce the viability of AML [12], and it is being evaluated in a phase 1 clinical trial for patients with myeloma and lymphoma [13].

Specifically for lymphoblastic leukemia, the effect of PTL has been evaluated by Diamanti, who demonstrated that this compound can induce apoptosis in CD34^+^CD19^−^ or CD34^+^CD7^−^ ALL cells, preventing the engraftment capacity in NSG models [2]. The effects of DMAPT or PU-H71 have not been yet explored. The aims of this work were to evaluate the effect of PTL, DMAPT, and PU-H71 in B-ALL cell lines as well as different hematopoietic populations from ALL pediatric patients at diagnosis

## 2. Results

### 2.1. Effect of PLT, DMAPT, and PU-H71 in ALL Cell Lines

**Figure 1:**
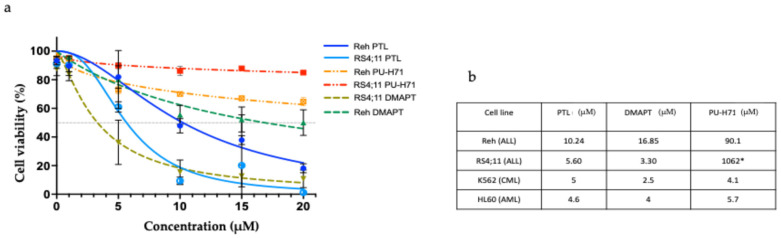
PTL, DMAPT, and PU-H71 reduced viability of B-ALL cell lines. Reh and RS4;11 leukemic cell lines were cultured for 48 h at different concentrations of PTL DMAPT and PU-H71. The results in (**a**) correspond to the viability of each cell line, and the table in (**b**) represents the IC50. K562 and HL60 cell lines were used as a positive control. All experiments were performed three times in triplicate. * This concentration was calculated due to its low effect at the analyzed concentrations.

To analyze the effect of the compounds in B-ALL cells, we first exposed RS4;11 and Reh cell lines to different concentrations (from 1 to 20 µM) of PTL, DMAPT, or PU-H71 for 48 h. Then, cell viability was evaluated by flow cytometry using 7AAD. The results in Figure 1a show that viability of both cell lines was reduced by the treatment with the compounds in a dose-dependent manner, emphasizing the RS4;11 cells as being particularly sensitive. PU-H71 had a lesser effect than PTL and DMAPT, which can be seen at the inhibitory concentration 50 (IC50) (Figure 1b). These IC50 values for B-ALL cell lines are akin to a previous report for CML and AML conducted by our lab [10], with the difference that for B-ALL cell lines, PU-H71 IC50 had to be calculated due to its low effect at the analyzed concentrations.

### 2.2. Primary B-ALL Cell Characterization

**Figure 2:**
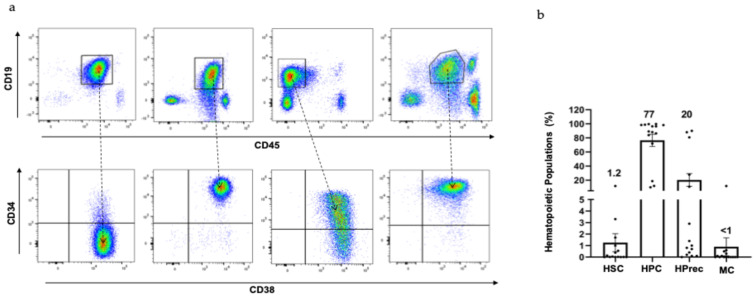
Primary B-ALL samples were highly heterogeneous. Mononuclear cells from primary ALL pediatric patients were obtained and stained by flow cytometry analysis. (**a**) shows a representative dot plot from different populations associated with CD19 and CD45 expression, and leukemic blast population (CD19^+^CD45^−/med^) is indicated within the square. Also, a representative dot plot with the presence of hematopoietic compartment [stem cells (HSC; CD34^+^CD38^−^) hematopoietic progenitor cells (HPC; CD34^+^CD38^+^), hematopoietic precursor cells (Hprec; CD34^−^CD38^+^) cells, and mature cells MC; CD34^−^CD38^−^)] contained in the leukemic blast is shown. The average percentage of each population in all analyzed samples (n = 15 by duplicate by each primary sample) is included in (**b**).

Before evaluating the effect these compounds have on primary samples and considering the heterogeneity for B-ALL described in the literature, we decided to characterize all samples included in this study. For this, bone marrow plasma as well as bone marrow mononuclear cells (MNC) were obtained by centrifugation or density gradient, respectively. MNC were stained with antibodies against CD19, CD45, CD34, and CD38 to evaluate them by flow cytometry and identify the blast population content as well as primitive hematopoietic cell presence. As was expected, the results in Figure 2a show that there was an enormous heterogeneity between samples, showing up to four different populations associated with CD19 and CD45 expression. However, in all cases, leukemic blast subpopulation defined by the CD19^+^CD45^−/med^ CD19^+^CD45^−/intermediate^ immunophenotype was the most abundant. In some samples, we also observed cells with high or null CD45 expression, and we considered that they could represent normal hematopoietic cells or bone marrow non-hematopoietic populations, respectively.

Considering that B-ALL has mostly been reported as a progenitor or precursor hematopoietic cell disease, we decided to analyze the content of precursor (CD34^−^CD38^+^), progenitor (CD34^+^CD38^+^), or stem (CD34^+^CD38^−^) cells in the blastic population (CD19^+^CD45^−/intermediate^) by flow cytometry (Figure 2a). As shown in Figure 2b, we detected that 77% of samples corresponded to blasts that also express the progenitor immunophenotype, while the remaining 20% were associated with the precursor immunophenotype. In all cases, the stem cell population was only represented by 1.2%. These results confirm that B-ALL is a heterogenous progenitor or precursor hematopoietic disease, raising the need to know the impact of these phenotypic differences on the treatment response.

**Figure 3:**
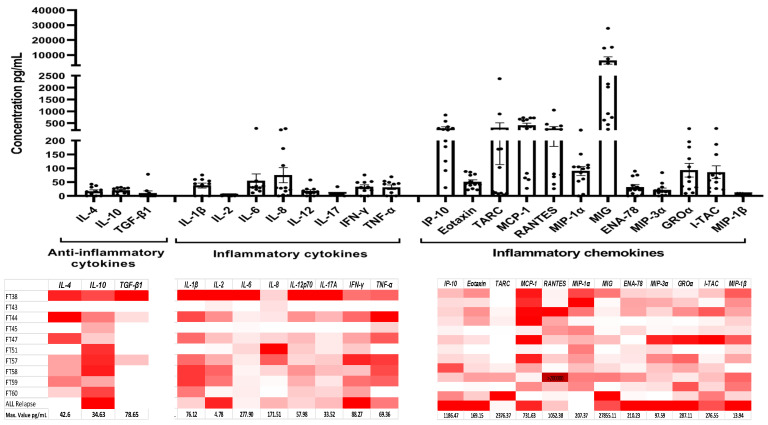
Primary B-ALL samples were surrounded by an inflammatory microenvironment. Cytokine and chemokine concentration (pg/mL) in B-ALL bone marrow plasma was evaluated by LEGENDplex^TM^ immunoassay at diagnosis. The data are expressed as mean ± SEM of all analyzed samples (n = 13 by duplicate by each primary sample at diagnosis and 1 sample at relapse). The maximum value of each molecule is indicated at the bottom of the heat map.

Additionally, the analysis of bone marrow plasmatic fraction (Figure 3) showed that, at the time of diagnosis, all samples were surrounded by an inflammatory microenvironment, inferred by the high concentration of inflammatory cytokines and chemokines. Interestingly, although these are secreted at different concentrations depending on the sample (according to the heat map), they are mostly absent in plasma from umbilical cord blood samples (Figure S1). However, the highest concentration of chemokine was observed in plasma from a relapsed B-ALL patient.

### 2.3. Leukemic Cell Elimination

With the goal to eliminate leukemic populations and considering that PTL and DMAPT have anti-inflammatory and antitumor action, MNC from each primary B-ALL sample were cultured in presence or absence of 5 µM of PTL, DMAPT, or PU-H71; 48 h later, apoptosis (early and late) was assessed using flow cytometry. This compound concentration was selected for two reasons: (1) because these primary (MNC or CD34^+^lin^−^) cells cannot remain in culture for more than five days in the absence of any additional stimulus and (2) because this concentration is near the IC50 for B-ALL cell lines (Figure 1) and because in our previous studies with acute and chronic myeloid leukemias, 5 µM and 24–48 h of stimulation ware an efficient way to induce cell death in bulk or stem cell populations [10].

**Figure 4:**
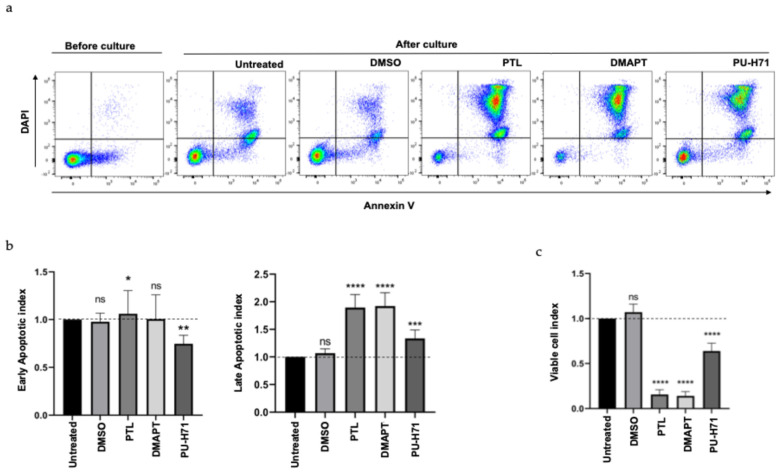
PTL, DMAPT, and PU-H71 induced apoptosis cell death in primary B-ALL cells. MNC from B-ALL samples were cultured in the presence or absence of 5 µM of PTL, DMAPT, or PU-H71 for 48 h, and the apoptosis index was analyzed by flow cytometry with DAPI and Annexin V stain. (**a**) shows a representative dot plot with the level of apoptosis before and after culture in the presence or absence of each treatment. The average of early (Annexin V^+^DAPI^−^) or late apoptosis (Annexin V^+^DAPI^+^) (**b**) as well as viable cells (**c**) in all primary B-ALL samples analyzed (n = 15, by duplicate by each primary sample) is shown. Apoptosis index was determined by dividing the cell number of dead cells in PTL-, DMAPT-, or PU-H71-treated cells by the frequency of cell number detected in the untreated group (considered as 1 and indicated with a horizontal line). A similar strategy was used to determine the viable cell index. The significance between apoptosis index in UT and treated cells was determined by the Mann–Whitney test (* *p* < 0.03; ** *p* < 0.0038, *** *p* < 0.0009, **** *p* < 0.0001, ns means not significant).

Figure 4a is a representative dot plot where the early (DAPI^−^AnnexinV^+^) or late (DAPI^+^AnnexinV^+^) apoptotic cells after treatment are indicated. During the 48 h of cell culture, there was an increase in apoptosis level, both with or without the compound (this was considered as basal apoptosis). Nevertheless, with all the compounds, the level of apoptosis was increased, observing a minimal early apoptosis with PTL treatment; however, late apoptosis increased significantly after treatment with every compound (Figure 4b), finding a greater effect with PTL or DMAPT in relation with PU-H71. These cell death results correlated with the reduction in viable cells (DAPI^−^AnnexinV^−^) observed in Figure 4a,c, indicating that these compounds can eliminate B-ALL cells

**Figure 5:**
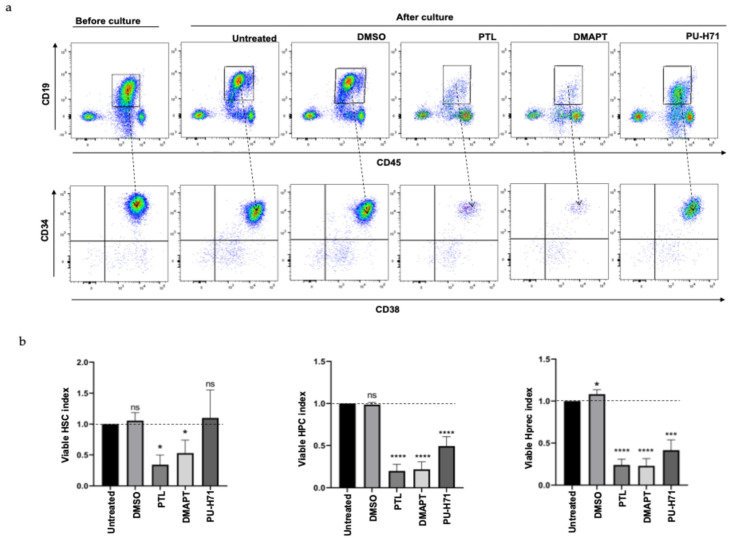
PTL DMAPT and PU-H71 eliminated blast, progenitor, and precursor cells from B-ALL. The viable remaining population before and after culture with 5 µM of PTL, DMAPT, or PU-H71 for 48 h was analyzed according to CD19, CD45, CD34, and CD38 expression. (**a**) shows a representative dot plot of the blast (CD19^+^CD45^−/med^) population as well as the stem (CD34^+^CD38^−^), progenitor (CD34^+^CD38^+^), or precursor (CD34^−^CD38^+^) content within this. (**b**) shows the average (n = 15, by duplicate by each primary sample) of viable cells in progenitor or precursor fraction after each treatment. Viable cell index was determined by dividing the cell number of viable cells in PTL-, DMAPT-, or PU-H71-treated cells by the cell number of viable cells detected in the untreated group (considered as 1 and indicated with a horizontal line). The significance between the apoptosis index in UT and treated cells was determined by the Mann–Whitney test (* *p* < 0.03; *** *p* < 0.0002, **** *p* < 0.0001, ns means not significant).

Additionally, using the strategy for immunophenotyping previously described, the characteristics in the remaining viable population were analyzed. The representative dot-plot shows a significant decrease in the total viable blasts CD19^+^CD45^negative/low^ as well as all hematopoietic subpopulations contained within them (Figure 5a). Once again, the data showed a greater reduction in viable cells after using PTL and DMAPT, compared to PU-H71, which had a minor effect. This observation was consistent in all analyzed samples (Figure 5b) where a reduction in cells with stem, progenitor, or precursor immunophenotype cells was observed after each treatment. 

Finally, in the primary samples where the initial MNC number was greater than 100 × 10^6^, the CD34^+^lin^−^ progenitor cells were enriched using immunomagnetic selection. From this assay, between 0.2 and 0.6% CD34^+^lin^−^ cells were recovered, showing a reduced cell number in relation to that previously reported by us in normal or myeloid leukemias [14].

**Figure 6:**
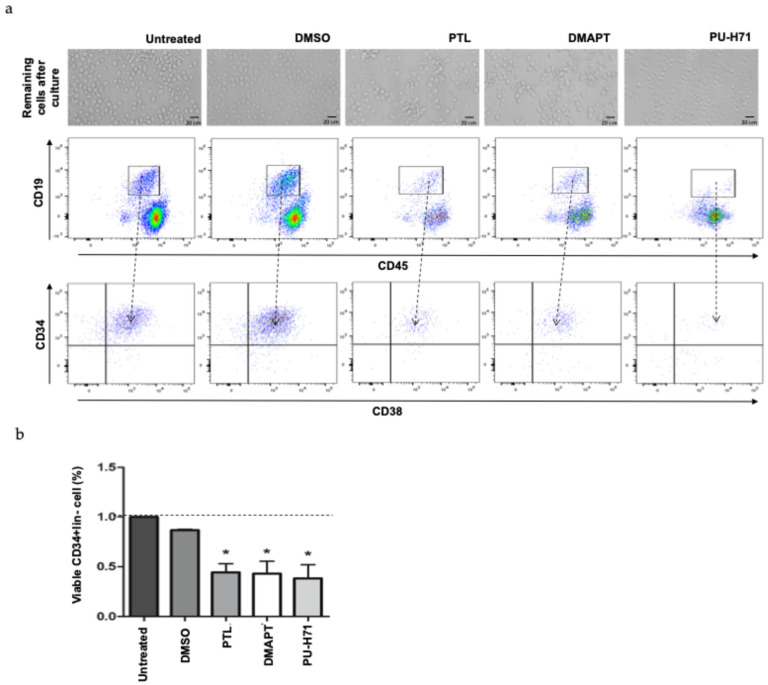
PTL, DMAPT, and PU-H71 reduced cell viability in enriched CD34^+^lin^−^ from B-ALL. CD34^+^lin^−^ cells were enriched by immunomagnetic selection and cultured in the presence or absence of 5 µM of PTL, DMAPT, or PU-H71 for 48 h, and viable cells were analyzed. (**a**) A representative photograph and dot plot of alive cells after each condition culture and their immunophenotype (blast CD19^+^CD45^−/med^, progenitor CD34^+^CD38^+^, or precursor CD34^−^CD38^+^) are shown. (**b**) The percentage of viable cells in CD34^+^lin^−^ enriched cells (n = 3, by duplicate by each primary sample) after each treatment is shown. Percentage was determined by considering 100% as the number of viable cells in untreated culture (indicated with a horizontal line) by the cell number obtained after PTL, DMAPT, or PU-H71 treatment. The significance between viable index in untreated and treated cells was determined by Student’s *t*-test (* *p* < 0.05).

To know the effect of the compounds on these progenitor cells, CD34^+^lin^−^ cells were cultured for 48 h in the presence or absence of PTL, DMAPT, or PU-H71 at 5 µM, after which, the remaining population was analyzed. Figure 6a shows a representative image of each cell culture and the reduction in cell number as well as morphological changes after treatment with these compounds was evident. Additionally, the immunophenotypic evaluation indicated that cultured progenitor enriched cells still retained cells with the blast (CD19^+^CD45^negative/low^) immunophenotype but were reduced when primitive cells were exposed to the compounds. Furthermore, a considerable reduction in primitive CD34^+^lin^−^ was also observed. Interestingly, the elimination of leukemic cells from this population was higher with PU-H71 treatment.

When the number of colony-forming cells was analyzed, no colony was detected before or after any treatment, suggesting that the cultured conditions are not appropriate to evaluate the functional capability of cells with a lymphoid origin. Importantly, myeloid CFCs were also absent in the blast fraction in B-ALL patients, suggesting a numerical reduction of myeloid progenitor that could have been displaced by the lymphoid leukemic growth.

**Figure 7:**
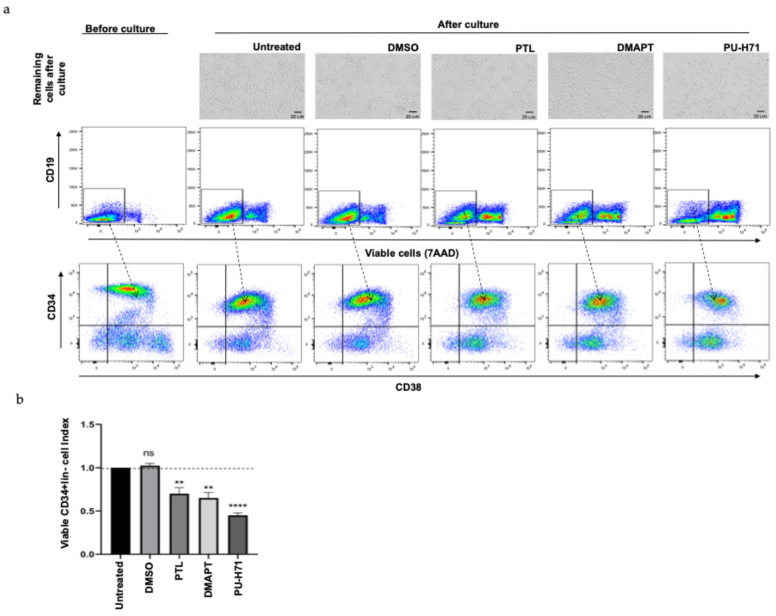
PTL, DMAPT, and PU-H71 had a minimal effect on the viability of normal hematopoietic cells. Enriched CD34^+^lin^−^ from mobilized peripheral blood samples was cultured in the presence or absence of 5 µM of PTL, DMAPT, or PU-H71 for 48 h, and the presence of viable hematopoietic stem cells (CD34^+^CD38^−^), hematopoietic progenitor cells (CD34^+^CD38^+^), hematopoietic precursor cells (CD34^−^CD38^+^), and mature cells (CD34^−^CD38^−^) was analyzed by flow cytometry. (**a**) A representative photograph and dot plot of each culture and hematopoietic population are shown. The percentage of viable cells in total CD34^+^lin^−^ enriched cells (n = 3, by duplicate by each primary sample) after each treatment is shown (**b**). The index of viable cells in CD34^+^lin^−^ enriched cells after each treatment is shown. Index was determined by the cell number of viable cells in PTL-, DMAPT-, or PU-H71-treated cells by the cell number of viable cells detected in untreated culture (indicated with horizontal line). The significance between viable index in untreated and treated cells was determined by Student’s *t*-test test (** *p* < 0.003, **** *p* < 0.0001, ns means not significant).

On the other hand, contrasting with leukemic cells, when the evaluation of these compounds was performed with normal CD34^+^lin^−^ cells from mobilized peripheral blood, the results in the photographs and cytometric analysis (Figure 7a) showed a minor effect in the reduction of the viability of total population after PTL or DMAPT treatment (Figure 7b), compared with the effect observed in LLA cells (showed in Figure 5b) where the reduction was at least five times less. Again, we observed that PU-H71 reduced normal cells in a similar way to leukemic hematopoietic progenitor or precursor cells (Figure 7b). Importantly, no effect of PTL and DMAPT were observed in primitive hematopoietic populations related with CD34^+^ and CD38 expression, as is observed in the dot plots of Figure 7a.

Considering that the effect of B-ALL cells are immersed in a dynamic microenvironment constituted by different non hematopoietic cells, we analyzed the effect of three compounds in normal endothelial and mesenchymal stem cells and found that PTL and DMAPT did not have any effect on these normal cell types. Instead, PU-H71 had a minimal effect in reducing their viability (Figure S2).

All these results demonstrated that B-ALL cells (blast, progenitor, and precursor cells) can be eliminated with the use of 5 µM of PTL and DMAPT, which has a minimal effect on normal hematopoietic cells and microenvironmental populations. Furthermore, this effect is independent of the clinical characteristics at diagnosis of each patient, which suggests a generalized effect on pediatric B-ALL cells.

## 3. Discussion

ALL is the most common pediatric cancer, with an incidence rate of 10–45 cases per million children, and it is associated with adverse clinical outcome related to poor response to treatment, toxicity, and high mortality [15]. Biologically, this disease has been related to the presence of LIC that appears to not be eliminated by the different treatments due to them being able to remain within favorable microenvironments and in some cases remain in a non-proliferative status [10,16,17]. This scenario creates the necessity to search for new molecules that allow the elimination of bulk leukemic population as well as more primitive hematopoietic leukemic cells that may include LIC. With this goal in mind, the effect of PTL, DMAPT, and PU-H71 in B-ALL primary cells was assessed in this work.

The results showed that primary pediatric B-ALL represents a highly heterogeneous disease where each patient has a particular immunophenotypic and inflammatory context. Interestingly, we notice that samples from primary pediatric patients could contain hematopoietic cells with variable expressions of CD19 and CD45 antigens. This CD45 expression has been previously reported by Cario and co-workers [18] who found a correlation between CD45 expression and initial white blood cell count as well as poor response to prednisone treatment. In the same sense, the expression of CD19 is related with the resistance to blinatumomab CAR T cell therapy, since there are 10–20% of epitope loss CD19 locus [19]. This heterogeneity also has been reported for the expression of CD34 and CD38, finding that the presence of CD34-positve CD38 dim/positive blastic is associated with poor response to therapy and increase in relapse, an event that appears to be related to migration and cell adhesion [20].

Related to the above, our results indicate that BM leukemic plasma is enriched with chemokines such as CXCL10 (IP10), CXCL9 (MIG), and CXCL11 (I-TAC), which also have been associated with migration, tumor growth, metastasis, and poor prognosis in different tumors [21]. Moreover, inflammatory cytokines, such as IL1b, IL6, TNFa, and INF, were increased, and this relates to a previously described effect by Vilchis et al. [22], where an increase in cytokine production and hematopoietic growth factors is a result of culturing B-ALL MNC for 24 h. These results indicate that B-ALL blast cells are growing within an inflammatory microenvironment that also has been associated with leukemic maintenance.

Adding to the above, our data show that the blast cells analyzed in this work correspond to progenitor (77%) or precursor (20%) cells. This high percentage of samples with progenitor immunophenotype is in line with a recent report from Romo-Rodriguez et al. [23], who produced an extensive description of leukemic pediatric patients from different regions of Mexico. Additionally, our data show that at diagnosis, there is a reduction in populations with stem cell immunophenotype as well as in CD34^+^lin^−^ post-enrichment. This observation was previously reported by Demanou-Peylin et al. [24], who detected a reduction in CD34^+^CD38^−^ cells using flow cytometry. This reduced number of normal primitive populations inside the inflammatory microenvironment in B-ALL could be related to a failure to restore the normal hematopoiesis and in consequence the leukemic permanence.

In this sense and with the goal to eliminate leukemic cells, we cultured B-ALL MNC in the presence or absence of 5 µM of PTL, DMAPT, or PU-H71 and noticed that all compounds reduced viable population at the same time of inducing late apoptotic cell death. A similar result has been reported by Diamanti and coworkers (2018) [25], who showed an apoptotic cell death induction in bulk ALL samples after treatment with 10 µM of PTL, and also, like ourselves, they reported no effect in normal bone marrow samples. Interestingly, the effect of this molecule is reduced in sorted CD34^+^CD19^−^ B-ALL cells. This last result showed a difference with our work since we noticed a reduction in the hematopoietic population related with immunophenotypes for stem (CD34^+^CD38^−^), progenitor (CD34^−^CD38^+^), precursor (CD34^+^CD38^+^), and primitive enriched (CD34^+^lin^−^) cells after PTL and DMAPT treatment. This last compound had not been analyzed in this type of sample and could represent a good therapeutic option since it has a higher solubility and bioavailability. Also, DMAPT has minimal effects in normal hematopoietic populations, as we observed in this work and previous reports [10]. It is important to comment that the phenotypes analyzed in this paper are related to normal hematopoietic subpopulations; however, we have to consider that in acute leukemias, there are important phenotypical alterations. Specifically for ALL, it has been reported that there is a particular population (CD34^+^CD38^−/low^CD19^+^ cells) that is not detectable in normal bone marrow, and it is responsible for initiating ALL in transplanted mice [26]. This rare population could include the hematopoietic subpopulations analyzed in this study. It is also possible that this could coexist with the normal residual primitive fraction, since it has been described that normal stem cells are still present in patients, even when the leukemia is active and bone marrow is invaded by blasts [24].

Regarding PU-H71, we observed that although it is less efficient in reducing cell viability in comparison with PTL or DMAPT, it is able to reduce all leukemic analyzed populations. This result is like that observed for another competitive inhibitor to HSP90, NVP-BEB800, which reduces viability of cell lines in bulk, and primitive primary populations from B-ALL and also improves the survival of B-ALL in mice models [27]. In a similar way, Alvespimycin and Celastrol reduce bulk and sorted CD34^+^CD19^+^, CD34^+^CD19^−^, and CD34^+^ cells from B-ALL primary samples [25], highlighting a potential therapeutic use since these compounds do not show a toxic effect in normal cord blood or HSC. In contrast, PU-H71 significantly reduces the viability of CD34^+^lin^−^ from normal peripheral blood and endothelial cells; therefore, its therapeutic use must be taken with caution.

It is important to notice that although there was an important reduction in leukemic cell viability in all samples analyzed, a small cell number remained after each treatment, and it was possible that this population may include LIC with CD34^+^CD38^+^CD19^+^ or CD34^+^CD38^−^CD19^+^ cells, since a similar observation was made in 10 primary samples of T-ALL cells treated with PTL, where this cell resistance was associated with the soluble molecules produced by mesenchymal stem cells in the microenvironment [28]. In this sense, our results showed a high concentration of inflammatory molecules in the bone marrow plasma from patients, indicating a pro-inflammatory microenvironment that has been related with leukemic maintenance and LIC permanence. The presence of LIC in different immunophenotypical populations has also been reported by Kong et al. and Jiang et al. Kong et al. showed that CD34^+^CD38^+^CD19^+^ or CD34^+^CD38^−^CD19^+^ cells from three B-ALL pediatric samples were able to develop B-ALL in experimental mice, while Jiang and colleagues showed that CD34^+^CD38^−^, CD34^+^CD38^+^, and CD34^−^CD38^+^ from B-ALL adult patients could reconstitute B-ALL in a mouse model [3,29]. According to this information, it is imperative to find new strategies that allow the elimination of all leukemic populations, including those with stemness or leukemia-initiating characteristics.

In this way, this study represents an approximation to leukemic elimination, since PTL and DMAPT are able to eliminate in vitro different B-ALL populations (including LIC), and although minimal effects were observed in normal populations, the results showed here justify the need to carry out additional studies in which microenvironmental components can be included. Furthermore, it is possible that greater effects of PTL and DMAPT could be obtained in combination with other antileukemic agents. Finally, it is important to consider future in vivo approaches with the intention to evaluate the possible use of these molecules as therapeutic agents.

## 4. Materials and Methods

### 4.1. Cell Lines and Primary Samples

The cell lines RS4;11 and Reh from B-cell lineage ALL were purchased from the ATTC cell collection. The cells were cultured in RPMI 1640 medium (ATCC, Manassas, VT, USA) supplemented with 10% FBS (ATCC, Manassas, VT, USA) and 1% penicillin–streptomycin (Gibco, Thermo Fisher Scientific, Waltham, MA, USA).

Fifteen primary samples from the bone marrow (BM) of pediatric patients with B-ALL at diagnosis and without treatment were obtained according to the Ethics, Biosafety, and Research Committee of Instituto Mexicano del Seguro Social after written informed approval and consent from patients and parents (Registry R-2021-785-025). Clinical information about each patient is included in a Supplementary Table S1.

As normal control cells, we used hematopoietic cells from mobilized peripheral blood (from healthy young donors) and umbilical cord blood. Normal mesenchymal stem cells and normal endothelial cells were also obtained and characterized according to previous reports [30,31,32].

### 4.2. Cell Enrichment

Bone marrow mononuclear cells (MNC) from primary samples were isolated with Lymphoprep^TM^ (Stem Cell Technologies Inc., Vancouver, BC, Canada) gradient separation. CD34^+^lin^−^ cells were enriched using negative selection with the StemSep^TM^ system (Stem Cell Technologies Inc., Vancouver, BC, Canada). MNC were briefly incubated with an antibody cocktail (CD2, CD3, CD14, CD16, CD19, CD24, CD56, CD66b, and glycophorin A) for 15 min followed by incubation with a magnetic colloid. The enriched subpopulation was collected and cultured in serum-free expansion medium (Stem Span^TM^, Stem Cell Technologies Inc., Vancouver, BC, Canada) supplemented with 10 ng/mL of SCF, TPO, Flt-3L, IL-6, IL-3, G-CSF, and GM-CSF (PeproTech, Trenton, NJ, USA).

### 4.3. Compounds and Antibodies

PTL, DMAPT, and PU-H71 were kept at 40 mmol/L at −20 °C in DMSO. Different concentrations were used to calculate the inhibitory concentration 50 (IC50) according to Hill coefficients [33] using GraphPad Prism version 10.2. The experiments with primary samples were performed at 5 µM for each compound.

For ALL cell characterization, the next panel of antibodies was used: anti-human CD19-APC/Cyanine7 (Clone HIB19, 650 nm excitation/785 nm emission; Biolegend. San Diego, CA, USA), anti-human CD45-FITC (Clone HI30, 495 nm excitation/519 nm emission, Biolegend. San Diego, CA, USA), anti-human CD45-PE (Clone HI30, 565 nm excitation/578 nm emission, Biolegend. San Diego, CA, USA), anti-human CD45-APC (Clone QA21A24, 594 nm excitation/519 nm emission, Biolegend. San Diego, CA, USA), anti-human CD34-PE/Cyanine7 (Clone 561, 488 nm excitation/778 nm emission, Biolegend. San Diego, CA, USA), and anti-human CD38-APC (Clone HIT2/HB-7, 494 nm excitation/660 nm emission, Biolegend, San Diego, CA, USA). Additionally, for apoptosis and viability evaluation, Annexin V-FITC (494 nm excitation/519 nm emission, BD biosciences. New York, NY, USA), 7AAD (546 nm excitation/647 nm emission, Invitrogen, Waltham, MA, USA), DAPI (359 nm excitation/465 nm emission, Invitrogen), and Ghost Dye Red 780 (633 nm excitation/780 nm emission, TONBObiosciences. San Diego, CA, USA) were used.

In all cases, a minimum of 30,000 events of individual cells (SSC-A vs. SSC-H) was acquired. Hematopoietic subpopulations were analyzed from the total percent of cells, while viability and apoptosis were calculated from the total cell number.

### 4.4. Cultured in the Presence of PTL, DMAPT, and PU-H71

Cell lines as well as primary samples (MNC or CD34^+^lin^−^) from ALL or NBM were cultured at a density of 10^5^ cell per mL in 48-well plates. For MNC, Reh, and RS4;11 cell lines, RPMI 1640 medium supplemented with 10% FBS and 1% penicillin–streptomycin was used. CD34^+^lin^−^ cells were cultured in stem spam in medium in the presence of 10 ng/mL of SCF, TPO, Flt-3L, IL-6, IL-3, G-CSF, and GM-CSF. Cultures were added with the indicated concentration of PTL, DMAPT, or PU-H71 as well as negative controls and maintained in a humidified incubator at 5% CO_2_ and 37 °C for 48 h until respective evaluation.

### 4.5. Cell Viability and Colony-Forming Cell Content

Cell viability was evaluated by trypan blue exclusion or by flow cytometry using 7AAD and subsequently compared with control without treatment.

The presence of progenitor cells capable of forming colonies (CFC) was determined according to previously described methods [14]. Around 50,000 MNC or 3000 CD34^+^lin^−^ cells recovered from each culture (control or treated with PTL, DMAPT, or PU-H71) were sub-cultured for 14 days on a complete methylcellulose medium (MethoCult^TM^ H4434, Stem Cell Technologies Inc., Vancouver, BC, Canada). CFC were scored using an inverted microscope under 10×, and colony number was normalized with the quantified CFC for control without treatment.

### 4.6. Cell Death

Cell death index in MNC or CD34^+^lin^−^ cells was analyzed by flow cytometry (FACS Verse^TM^) using Annexin V (BD Bioscience. New York, NY, USA) and DAPI (BD Bioscience. New York, NY, USA). The apoptotic index was determined by calculating the ratio of cell death (total or early) in cultures treated with PTL, DMAPT, or PU-H71 to the cell death observed in untreated cultures (considered as 1).

### 4.7. Secretion of Soluble Components

The plasma from each sample was recovered, centrifuged, and stored at −70 °C until evaluation. The analysis was performed with plasma from B-ALL primary samples uL using LEGENDplex immunoassay (Biolegend 740930 and 740985, San Diego, CA, USA), according to the supplier instructions using FACS Aria II (BD Bioscience, New York, NY, USA). Data are reported in pg/mL using Qognit Free Software (https://legendplex.qognit.com/user/login?next=home, Biolegend, San Diego, CA, USA) [34].

### 4.8. Data Analysis and Statistics

The data obtained in the evaluation of the LEGENDplex^TM^ were analyzed with Qognit Software, while the flow cytometry data were analyzed with flowjo (9.00). The change index was always calculated in relation to the control without treatment (considered as one) using Prism (10.2.3). All data were analyzed with the prism software, and their distribution was calculated with the Kolmogorov–Smirnov test. In the case of results with parametric distribution, the statistical analysis used was Student’s *t*-test, while for the non-parametric distribution, the Mann–Whitney U test was used.

## Data Availability

Data is contained within the article and Supplementary Materials.

## Supplementary Materials

The following supporting information can be downloaded at https://www.mdpi.com/article/10.3390/ijms252111707/s1.

## References

[1] Terwilliger T., Abdul-Hay M. (2017). Acute lymphoblastic leukemia: A comprehensive review and 2017 update. Blood Cancer J..

[2] Diamanti P., Cox C.V., Moppett J.P., Blair A. (2013). Parthenolide eliminates leukemia-initiating cell populations and improves survival in xenografts of childhood acute lymphoblastic leukemia. Blood J. Am. Soc. Hematol..

[3] Kong Y., Yoshida S., Saito Y., Doi T., Nagatoshi Y., Fukata M., Saito N., Yang S.M., Iwamoto C., Okamura J. (2008). CD34+ CD38+ CD19+ as well as CD34+ CD38− CD19+ cells are leukemia-initiating cells with self-renewal capacity in human B-precursor ALL. Leukemia.

[4] Lee A.Q., Konishi H., Duong C., Yoshida S., Davis R.R., Van Dyke J.E., Ijiri M., McLaughlin B., Kim K., Li Y. (2022). A distinct subpopulation of leukemia initiating cells in acute precursor B lymphoblastic leukemia: Quiescent phenotype and unique transcriptomic profile. Front. Oncol..

[5] Inaba H., Pui C.H. (2021). Advances in the diagnosis and treatment of pediatric acute lymphoblastic leukemia. J. Clin. Med..

[6] Fregona V., Bayet M., Gerby B. (2021). Oncogene-induced reprogramming in acute lymphoblastic leukemia: Towards targeted therapy of leukemia-initiating cells. Cancers.

[7] Carlisi D., Lauricella M., D’Anneo A., De Blasio A., Celesia A., Pratelli G., Notaro A., Calvaruso G., Giuliano M., Emanuele S. (2022). Parthenolide and its soluble analogues: Multitasking compounds with antitumor properties. Biomedicines.

[8] Guzman M.L., Rossi R.M., Karnischky L., Li X., Peterson D.R., Howard D.S., Jordan C.T. (2005). The sesquiterpene lactone parthenolide induces apoptosis of human acute myelogenous leukemia stem and progenitor cells. Blood.

[9] Guzman M.L., Rossi R.M., Neelakantan S., Li X., Corbett C.A., Hassane D.C., Becker M.W., Bennett J.M., Sullivan E., Lachowicz J.L. (2007). An orally bioavailable parthenolide analog selectively eradicates acute myelogenous leukemia stem and progenitor cells. Blood.

[10] Flores-Lopez G., Moreno-Lorenzana D., Ayala-Sanchez M., Aviles-Vazquez S., Torres-Martinez H., Crooks P.A., Guzman M.L., Mayani H., Chávez-González A. (2018). Parthenolide and DMAPT induce cell death in primitive CML cells through reactive oxygen species. J. Cell. Mol. Med..

[11] Shrestha L., Bolaender A., JPatel H., Taldone T. (2016). Heat shock protein (HSP) drug discovery and development: Targeting heat shock proteins in disease. Curr. Top. Med. Chem..

[12] Zong H., Gozman A., Caldas-Lopes E., Taldone T., Sturgill E., Brennan S., Ochiana S.O., Gomes-DaGama E.M., Sen S., Rodina A. (2015). A hyperactive signalosome in acute myeloid leukemia drives addiction to a tumor-specific Hsp90 species. Cell Rep..

[13] Dunphy M. (2015). PET Imaging of Cancer Patients Using 124I-PUH71: A Pilot Study.

[14] Chávez-González A., Rosas-Cabral A., Vela-Ojeda J., González J.C., Mayani H. (2004). Severe functional alterations in vitro in CD34+ cell subpopulations from patients with chronic myeloid leukemia. Leuk. Res..

[15] Greaves M. (2018). A causal mechanism for childhood acute lymphoblastic leukaemia. Nat. Rev. Cancer.

[16] Fregona V., Bayet M., Bouttier M., Largeaud L., Hamelle C., Jamrog L.A., Prade N., Lagarde S., Hebrard S., Luquet I. (2023). Stem cell–like reprogramming is required for leukemia-initiating activity in B-ALL. J. Exp. Med..

[17] Ebinger S., Özdemir E.Z., Ziegenhain C., Tiedt S., Alves C.C., Grunert M., Dworzak M., Lutz C., Turati V.A., Enver T. (2016). Characterization of rare, dormant, and therapy-resistant cells in acute lymphoblastic leukemia. Cancer Cell.

[18] Cario G., Rhein P., Mitlöhner R., Zimmermann M., Bandapalli O.R., Romey R., Moericke A., Ludwig W.-D., Ratei R., Muckenthaler M.U. (2014). High CD45 surface expression determines relapse risk in children with precursor B-cell and T-cell acute lymphoblastic leukemia treated according to the ALL-BFM 2000 protocol. Haematologica.

[19] Orlando E.J., Han X., Tribouley C., Wood P.A., Leary R.J., Riester M., Levine J.E., Qayed M., Grupp S.A., Boyer M. (2018). Genetic mechanisms of target antigen loss in CAR19 therapy of acute lymphoblastic leukemia. Nat. Med..

[20] Modvig S., Wernersson R., Øbro N.F., Olsen L.R., Christensen C., Rosthøj S., Degn M., Jürgensen G.W., Madsen H.O., Albertsen B.K. (2022). High CD34 surface expression in BCP-ALL predicts poor induction therapy response and is associated with altered expression of genes related to cell migration and adhesion. Mol. Oncol..

[21] Hong Z., Wei Z., Xie T., Fu L., Sun J., Zhou F., Jamal M., Zhang Q., Shao L. (2021). Targeting chemokines for acute lymphoblastic leukemia therapy. J. Hematol. Oncol..

[22] Vilchis-Ordoñez A., Contreras-Quiroz A., Vadillo E., Dorantes-Acosta E., Reyes-López A., Quintela-Nuñez del Prado H.M., Venegas-Vázquez J., Mayani H., Ortiz-Navarrete V., López-Martínez B. (2015). Bone marrow cells in acute lymphoblastic leukemia create a proinflammatory microenvironment influencing normal hematopoietic differentiation fates. BioMed Res. Int..

[23] Romo-Rodríguez R., Zamora-Herrera G., López-Blanco J.A., López-García L., Rosas-Cruz A., Alfaro-Hernández L., Trejo-Pichardo C.O., Alberto-Aguilar D.R., Casique-Aguirre D., Vilchis-Ordoñez A. (2024). Subclassification of B-acute lymphoblastic leukemia according to age, immunophenotype and microenvironment, predicts MRD risk in Mexican children from vulnerable regions. Front. Oncol..

[24] Demanou-Peylin E., Blanc S., Da Costa Pereira T., Parietti V., Saintpierre B., Letourneur F., Souyri M., Domenech C. (2022). Novel insights into residual hematopoiesis from stem cell populations in pediatric B-acute lymphoblastic leukemia. Pediatr. Res..

[25] Diamanti P., Cox C.V., Moppett J.P., Blair A. (2018). Dual targeting of Hsp90 in childhood acute lymphoblastic leukaemia. Br. J. Haematol..

[26] Hong D., Gupta R., Ancliff P., Atzberger A., Brown J., Soneji S., Green J., Colman S., Piacibello W., Buckle V. (2008). Initiating and cancer-propagating cells in TEL-AML1-associated childhood leukemia. Science.

[27] Mshaik R., Simonet J., Georgievski A., Jamal L., Bechoua S., Ballerini P., Bellaye P.-S., Mlamla Z., de Barros J.-P.P., Geissler A. (2021). HSP90 inhibitor NVP-BEP800 affects stability of SRC kinases and growth of T-cell and B-cell acute lymphoblastic leukemias. Blood Cancer J..

[28] Ede B.C., Asmaro R.R., Moppett J.P., Diamanti P., Blair A. (2018). Investigating chemoresistance to improve sensitivity of childhood T-cell acute lymphoblastic leukemia to parthenolide. Haematologica.

[29] Jiang Z., Deng M., Wei X., Ye W., Xiao Y., Lin S., Wang S., Li B., Liu X., Zhang G. (2016). Heterogeneity of CD34 and CD38 expression in acute B lymphoblastic leukemia cells is reversible and not hierarchically organized. J. Hematol. Oncol..

[30] Dircio-Maldonado R., Flores-Guzman P., Corral-Navarro J., Mondragón-García I., Hidalgo-Miranda A., Beltran-Anaya F.O., Cedro-Tanda A., Arriaga-Pizano L., Balvanera-Ortiz O., Mayani H. (2018). Functional integrity and gene expression profiles of human cord blood-derived hematopoietic stem and progenitor cells generated in vitro. Stem Cells Transl. Med..

[31] Hernandez-Lopez R., Chavez-Gonzalez A., Torres-Barrera P., Moreno-Lorenzana D., Lopez-DiazGuerrero N., Santiago-German D., Isordia-Salas I., Smadja D., Yoder M.C., Majluf-Cruz A. (2017). Reduced proliferation of endothelial colony-forming cells in unprovoked venous thromboembolic disease as a consequence of endothelial dysfunction. PLoS ONE.

[32] Fajardo-Orduña G.R., Mayani H., Flores-Guzmán P., Flores-Figueroa E., Hernández-Estévez E., Castro-Manrreza M., Piña-Sánchez P., Arriaga-Pizano L., Gómez-Delgado A., Alarcón-Santos G. (2017). Human mesenchymal stem/stromal cells from umbilical cord blood and placenta exhibit similar capacities to promote expansion of hematopoietic progenitor cells in vitro. Stem Cells Int..

[33] Prinz H. (2010). Hill coefficients, dose–response curves and allosteric mechanisms. J. Chem. Biol..

[34] Torres-Barrera P., Moreno-Lorenzana D., Alvarado-Moreno J.A., García-Ruiz E., Lagunas C., Mayani H., Chávez-González A. (2022). Cell Contact with Endothelial Cells Favors the In Vitro Maintenance of Human Chronic Myeloid Leukemia Stem and Progenitor Cells. Int. J. Mol. Sci..

