# Supplementary material for: In Vitro Purging of Acute Lymphoblastic Leukemia (B-ALL) Cells with the Use of PTL, DMAPT, or PU-H71
Source: Int J Mol Sci. 2024 Oct 31;25(21):11707. doi: 10.3390/ijms252111707 (PMC11546800; doi:10.3390/ijms252111707)
Supplement: Supplementary file 1 [file ijms-25-11707-s001.zip › ijms-3266670-supplementary.pdf]

ID	Child´s sex	Child´s age at diagnosis (months)	WBC x103 at diagnosis in peripheral blood/ul	Translocations at diagnosis	Death	Relapse
FT-22	Male	190	2.00	Negative to ETV6/RUNX1, TCF3/PBX1, BCR/ABL1, MLL/AFF1	No	No
FT-28	Female	45	3.48	Negative to ETV6/RUNX1, TCF3/PBX1, BCR/ABL1, MLL/AFF1	No	Yes (bone marrow)
FT-30	Male	104	6.96	Negative to ETV6/RUNX1, TCF3/PBX1, BCR/ABL1, MLL/AFF1	No	No
FT-35	Female	89	4.72	Negative to ETV6/RUNX1, TCF3/PBX1, Positive to BCR::ABL1p190	No	No
FT-37	Male	199	1.96	Negative to ETV6/RUNX1, TCF3/PBX1, BCR/ABL1, MLL/AFF1	Yes (Septic shock)	No
FT-38	Female	38	42.72	Negative to ETV6/RUNX1, TCF3/PBX1, BCR/ABL1, MLL/AFF1	No	No
FT-43	Male	192	5.50	Negative to ETV6/RUNX1, TCF3/PBX1, BCR/ABL1, MLL/AFF1	No	No
FT-44	Male	94	5.00	Negative to ETV6/RUNX1, TCF3/PBX1, BCR/ABL1, MLL/AFF1	No	No
FT-45	Male	205	40.90	Negative to ETV6/RUNX1, TCF3/PBX1, BCR/ABL1, MLL/AFF1	No	No
FT-47	Female	82	51.43	Negative to ETV6/RUNX1, TCF3/PBX1, BCR/ABL1, MLL/AFF1	No	No
FT-55	Female	194	14.15	Negative to ETV6/RUNX1, TCF3/PBX1, BCR/ABL1, MLL/AFF1	No	No
FT-57	Male	189	11.11	Negative to ETV6/RUNX1, TCF3/PBX1, BCR/ABL1, MLL/AFF1	No	No
FT-58	Female	134	9.20	Negative to ETV6/RUNX1, TCF3/PBX1, BCR/ABL1, MLL/AFF1	No	No
FT-59	Female	60	1.64	Negative to ETV6/RUNX1, TCF3/PBX1, BCR/ABL1, MLL/AFF1	No	No
FT-53	Male	63	9.20	Negative to ETV6/RUNX1, TCF3/PBX1, BCR/ABL1, MLL/AFF1	Yes (Invasive mycosis)	No

Table S1. Clinical information of each patient included in this study

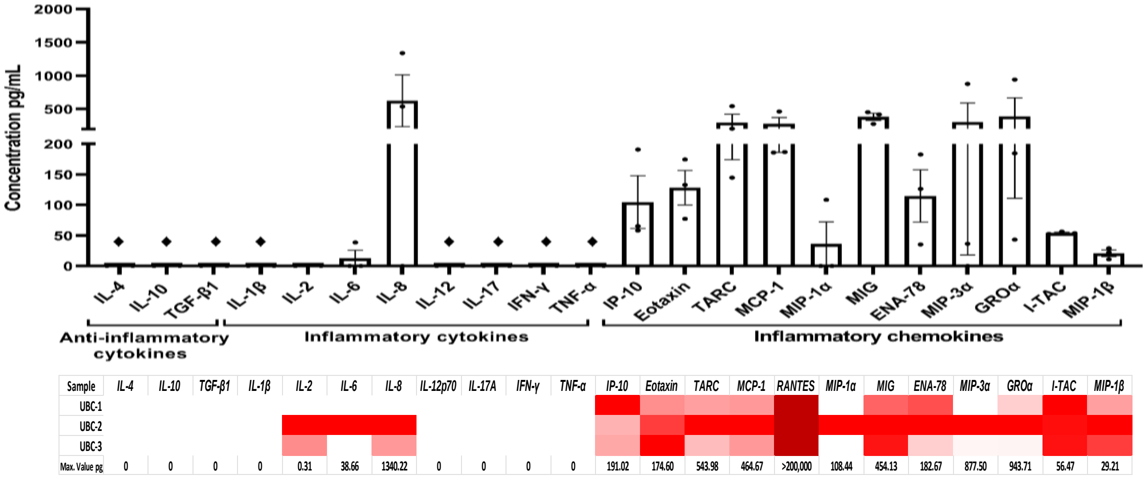

Figure S1. Concentration of inflammatory cytokines and chemokines in plasma from Umbilical Cord Blood is reduced in relation with B-ALL samples. Cytokine and chemokine concentration (pg/mL) in UCB samples were evaluated by milliplex immunoassay. The data are expressed as Mean ± SEM of all analyzed samples. The maximum value of each molecule is indicated at the bottom of the heat map and is significantly lower than detected in leukemic plasma samples.

Supplementary 2.

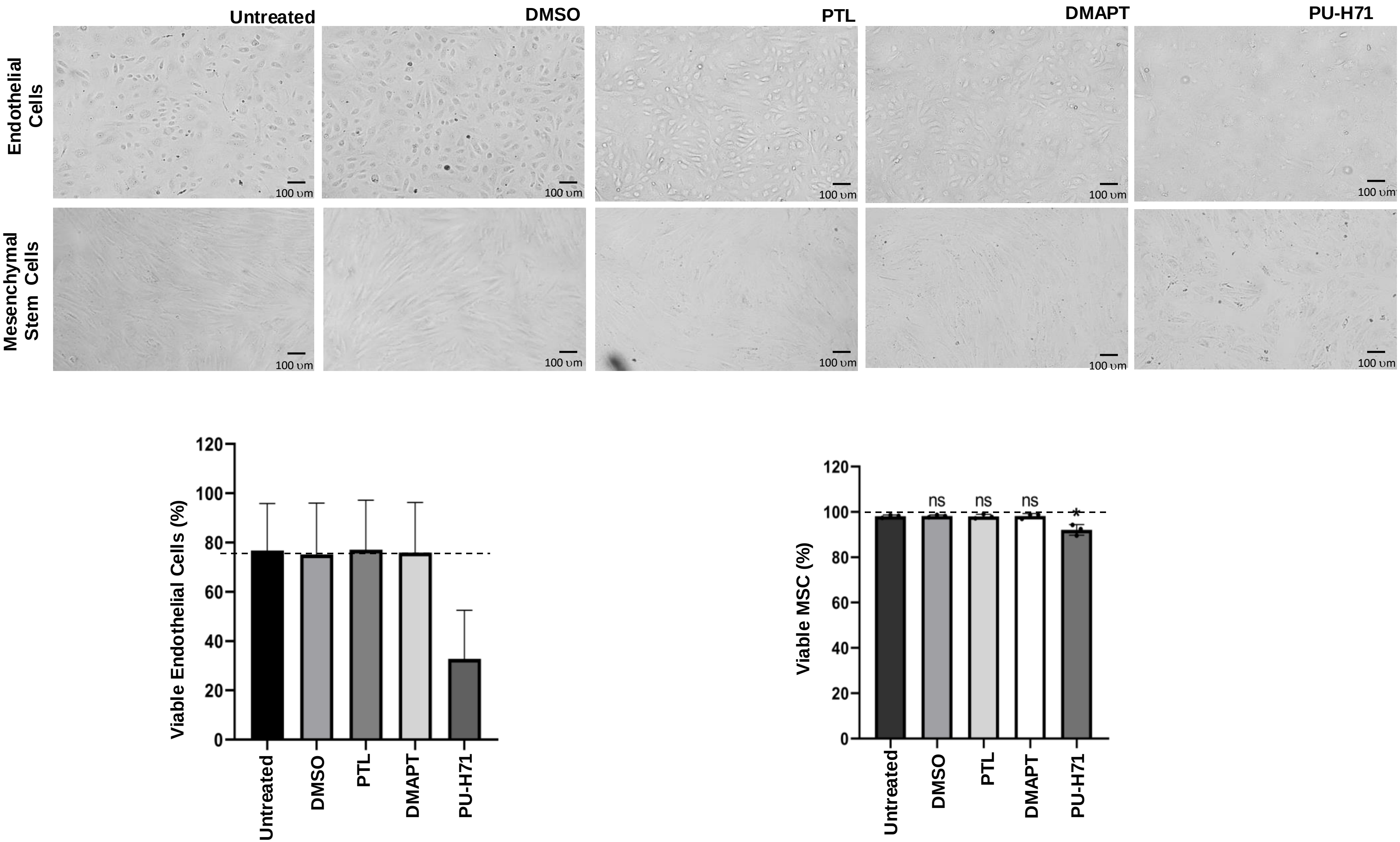

Figure S2. PTL, DMAPT and PU-H1 have a minimal effect in normal non hematopoietic cells
Endothelial and MSC were cultured in presence or absence of 5uM of PTL, DMAPT or PU-H71 and after 48 hours of culture and the viability was analyzed. A representative photograph and the percentage of viable cells after each treatment is shown. The percentage was determined to consider as 100% the number of viable cells in untreated culture (indicated with a horizontal line) by the number obtained after PTL, DMAPT or PUH71 treatment plot. Significance between viable index in untreated and treated cells was determined by t-student test ($p<0.05$).
